# Metagenomic Detection of Two Vientoviruses in a Human Sputum Sample

**DOI:** 10.3390/v12030327

**Published:** 2020-03-18

**Authors:** Fernando Lázaro-Perona, Elias Dahdouh, Sergio Román-Soto, Sonia Jiménez-Rodríguez, Carlos Rodríguez-Antolín, Fernando de la Calle, Alexander Agrifoglio, Francisco Javier Membrillo, Julio García-Rodríguez, Jesús Mingorance

**Affiliations:** 1 Servicio de Microbiología, Hospital Universitario La Paz, Idipaz, 28046 Madrid, Spain; fernandolazaroperona@gmail.com (F.L.-P.); elie.dahdouh@idipaz.es (E.D.); serromansoto@gmail.com (S.R.-S.); sonia23jr@gmail.com (S.J.-R.); juliogarciarodriguez@gmail.com (J.G.-R.); 2 Cancer Epigenetics Laboratory, Instituto de Genética Médica y Molecular (INGEMM), Hospital Universitario La Paz, 28046 Madrid, Spain; rodriguez.antolin.c@gmail.com; 3 Sección de Infecciosas and Unidad de Medicina Tropical y del Viajero, Servicio de Medicina Interna, Hospital Universitario La Paz, 28046 Madrid, Spain; fercalleprieto@gmail.com; 4 Unidad de Medicina Intensiva, Hospital Universitario La Paz, 28046 Madrid, Spain; alexander.agrifoglio@salud.madrid.org; 5 CBRN & Infectious Diseases Unit, Hospital Central de la Defensa Gómez Ulla, 28047 Madrid, Spain; fmemnov@oc.mde.es

**Keywords:** circoviruses, redondoviruses, metagenomics, respiratory disease

## Abstract

We used metagenomics to analyze one sputum sample from a patient with symptoms of a respiratory infection that yielded negative results for all pathogens tested. We detected two viral genomes that could be assembled and showed sequence similarity to redondoviruses, a recently described group within the CRESS-DNA viruses. One hundred sputum samples were screened for the presence of these viruses using specific primers. One sample was positive for the same two viruses, and another was positive for one of them. These findings raise questions about a possible role of redondoviruses in respiratory infections in humans.

## 1. Introduction

Metagenomic techniques are increasingly being used for the identification of potential pathogens in samples that test negatively in routine clinical assays [1,2,3]. The advantage of this approach is that it does not require a prior assumption about a target pathogen and may be particularly useful when the patient might have been exposed to uncommon pathogens. The use of clinical metagenomics has increased the discovery rate of new viruses, many of them still unclassified, not associated with any known disease, and therefore not targeted by diagnostic tests [4].

The use of rolling circle amplification techniques, which are known to preferentially amplify the circular rep-encoding single stranded DNA (CRESS-DNA) viruses, has revealed a wide variety of new CRESS-DNA viruses present in human samples, including respiratory samples, pericardial liquid, cerebrospinal fluid, stools, and blood [5]. CRESS-DNA viruses are among the smallest sized viruses (2–3 kb) that infect eukaryotic cells. They are highly diverse and ubiquitous and are frequently found in metagenomic analyses of soils, water, and animal samples [6]. The CRESS-DNA viruses usually encode a replication initiation protein (Rep) and a capsid protein (Cap). They have mutation rates similar to those of RNA viruses and high recombination rates that make classification a difficult task. To date, only six families of CRESS-DNA viruses are recognized by the International Committee on Taxonomy of Viruses (ICTV) [6], although an update including ten families has been approved (https://talk.ictvonline.org/files/proposals/animal_dna_viruses_and_retroviruses/m/animal_dna_ec_approved/9354). Among these, *Circoviridae*, *Bacilladnaviridae*, and *Smacoviridae* have been found to infect animals, although a few are known to cause serious illness in vertebrates, e.g., porcine circovirus-2 [7].

In this work, we report the detection by metagenomics of two human respiratory viruses in a sample from a patient with respiratory symptoms.

## 2. Materials and Methods

Total nucleic acids were extracted using the MagNA Pure Compact system (Roche Diagnostics GmbH, Mannheim, Germany). A fraction was treated with DNAse I to increase the RNA/DNA ratio and RNA was reverse transcribed. The cDNA was pooled with a fraction of untreated DNA, and amplified with the Illustra GenomiPhi V2 DNA Amplification Kit (GE Healthcare Biosciences, Madrid, Spain). Resulting products were fragmented with the Covaris m220 sonicator (Covaris, Woburn, MA, USA), a library was prepared according to the manufacturer’s instructions (NebNext Ultra Library Preparation Kit, New England BioLabs, Ipswich, MA, USA) and sequenced with the Illumina Miseq system using the MiSeq Reagent Kit v2 Micro, and 2 × 150 paired-end reads (Illumina, Foster City, CA, USA). TRIMMOMATIC-v0.32 [8] and STAR software [8] were used to eliminate adaptor sequences, short reads, and human reads using the Genome Reference Consortium Human Build 38 (GRCh38). The remaining reads (2,022,384) were classified using Taxonomer (www.taxonomer.com) and OneCodex (www.onecodex.com). Geneious v.10.1.3 (Biomatter Ltd., Auckland, New Zealand) was used for sequence analyses and primer design. Sequence phylogenetic analyses were done by the maximum likelihood method based on the JTT matrix-based model using MEGA X software [9]. Initial trees for the heuristic search were obtained by selecting the topology with greater log likelihood values after applying the neighbor-joining and BioNJ algorithms to a pairwise distance matrix estimated by a JTT model. A discrete Gamma distribution was used to model evolutionary rate differences among sites (5 categories (+G, parameter = 19,688)). The rate variation model allowed for some sites to be evolutionarily invariable ((+I), 0.52% sites).

Endpoint PCR amplifications were carried out in 50 µL volumes containing 10 µL 5× Buffer (Biotools, Madrid, Spain), and 1 µL of each dNTP (10 mM stock, Biotools, Spain), forward and reverse primers (10 mM stock, Merck KGaA, Darmstadt, Germany), and *Taq* polymerase (1 U/mL, Biotools, Spain). The thermocycling conditions were 94 °C for 2 min, followed by 45 cycles of 94 °C for 15 s, 55 °C for 30 s, and 72 °C for 1 min, and a final elongation step for 72 °C for 7 min. 

For Sanger sequencing, PCR products were treated with Exo-SapIT (Affimetrix, Santa Clara, CA, USA) and cycle sequencing was done with Big Dye 3.1 (Applied Biosystems, Foster City, CA, USA) according to the manufacturers’ protocols. 

qPCR reactions were performed in a CFX Connect Real-time system (BioRad, Spain) with the following mix: 6 µL of H_2_O, 10 µL of Power-Up SYBR Green Master Mix (Applied Biosystems, Foster City, CA, USA), 1 µL of each primer (10 mM stock, Sigma Life Sciences, Spain), and 2 µL of the sample DNA for a total volume of 20 µL. The thermocycling conditions for the primers targeting the conserved regions were an initial denaturation at 95 °C for 3 min, followed by 45 cycles of 95 °C for 15 s, 52 °C for 15 s, and 72 °C for 1 min. The conditions for the variant primers were an initial denaturation at 95 °C for 3 min, followed by 45 cycles of 95 °C for 15 s and 60 °C for 1 min. Both reactions were followed by a melting curve that went from 65 to 95 °C in 0.5 °C increments.

## 3. Results and Discussion

The case patient was a Spanish man that had traveled to Nepal. During the travel, he was in close contact with the rural environment and was bitten by mosquitoes and leeches. He had not eaten any raw food and did not report contact with rodents, cats, or other mammals. Except for a brief period of conjunctival erythema, he did not report any health-related events during travel. Three days after returning, he presented with a distempered sensation, shivering, and headache. Upon admission to Hospital Universitario La Paz (HULP), he developed respiratory distress, respiratory insufficiency, hypertransaminasemia, and thrombocytopenia, and was admitted to the intensive care unit. His condition evolved favorably, and he was discharged three weeks after admission. 

During the first few days of admission, blood, serum, urine, sputum, and nasopharyngeal samples were collected. Microbiological assays performed included bacterial and fungal cultures, serological tests for the detection of *Brucella* spp., *Borrelia* spp., *Leptospira* spp., hantavirus, dengue virus, *Coxiella* spp., *Mycoplasma* spp., *Chlamydia* spp., syphilis, HIV, hepatitis A/B/C viruses, cytomegalovirus, Epstein–Barr virus (EBV), and herpes virus. Additionally, legionella and pneumococcal antigen tests, and two molecular commercial kits targeting respiratory microorganisms (BioFire^®^ FilmArray^®^ Pneumonia Panel (bioMérieux, Marcy-l’Étoile, France) and Clart^®^ PneumoVir Panel (Genomica, Madrid, Spain)) were performed. All these assays yielded negative results. 

**Figure 1:**
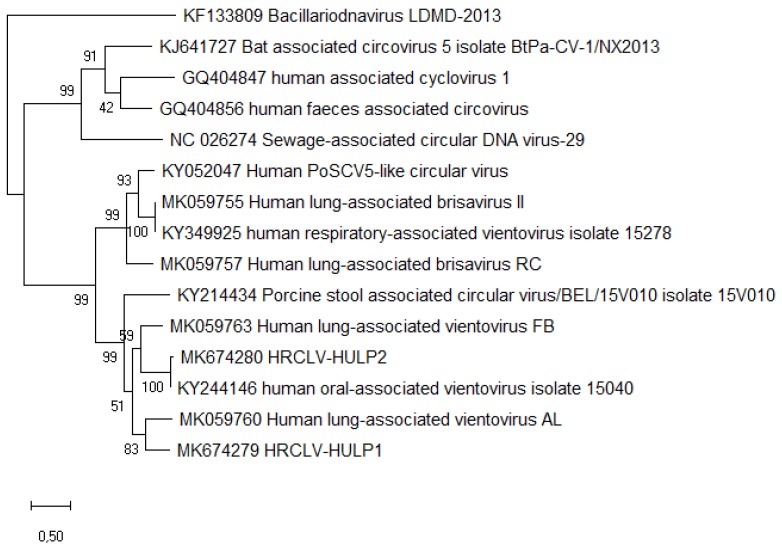
Molecular phylogenetic analyses of the Rep protein sequences of the two sequenced viruses compared with selected sequences from *Redondoviridae* and other close groups (*Circoviridae*, CRESSV2, and *Bacilladnaviridae*). The evolutionary history was inferred by using the maximum likelihood method based on the JTT matrix-based model. The tree with the highest log likelihood (−9456.60) is shown. The percentage of trees in which the associated taxa clustered together is shown next to the branches. Initial tree(s) for the heuristic search were obtained automatically by applying neighbor-joining and BioNJ algorithms to a matrix of pairwise distances estimated using a JTT model, and then selecting the topology with superior log likelihood value. The tree is drawn to scale, with branch lengths measured in the number of substitutions per site. Evolutionary analyses were conducted in MEGA X [9].

During the time in which the patient was admitted to HULP, a second case with similar symptoms and from the same travel group was admitted to Hospital Gómez Ulla (both hospitals are located in Madrid), suggesting the possibility of an infectious disease. Both patients had negative results in all the microbiological assays performed; therefore, a metagenomic approach was undertaken. A sputum sample from the case patient was obtained, centrifuged to remove human and bacterial cells, and total nucleic acids were extracted from the supernatant and sequenced with the Illumina Miseq system. After filtering the human reads, the 2,022,384 remaining reads were analyzed (Genbank Biosample accession number SAMN14178841). Bacterial reads included common oral bacteria (e.g., *Prevotella* spp.) and some species known to be common kit contaminants (e.g., *Shewanella* spp. and *Herbaspirillum* spp.) [10]. Viral species were mostly phages from oral bacteria, but 28 reads were classified as “porcine stool-associated circular virus 5” (PoSCV 5). A BLAST search on the NCBI database using these reads identified several matches belonging to small circular viruses: two porcine stool-associated viruses (accession numbers KJ433989 and KY214434) and three human respiratory tract associated viruses (KY052047, KY349925, and KY244146) [11]. Mapping all the non-human reads against one of these sequences (KY244146) yielded an assembly of 100% query cover and 99% nucleotide sequence identity to the reference sequence. The coverage in the capsid gene region was twice that of the replicase gene region, suggesting the presence of two variants of the virus in the sample. Visual analysis of the reads surrounding the replicase gene showed two slightly different sequences on each side. Primers for each putative variant were designed (Table S1) and used for endpoint PCR amplification and Sanger sequencing. The complete sequences of the two variants were obtained and called HRCLV-HULP1 (Genbank accession number MK674279) and HRCLV-HULP2 (Genbank accession number MK674280). HRCLV-HULP1 and HRCLV-HULP2 were 3052 and 3053 nucleotides in length, respectively. Both had the typical structure of the CRESS-DNA viruses, with two open reading frames (ORF), one encoding a helicase (Rep) and one encoding the capsid protein (Cap). A third ORF overlapping the capsid gene was detected that codes for a leucine-rich protein (34% Leu + Ile), similar to the ORF3 of the recently described *Redondoviridae* [12]. The two genomes had a small intergenic region of 178 and 175 nucleotides between the 5’ end of the Cap and Rep genes, with a potential stem loop sequence and a large intergenic region of 239 and 238 nucleotides between the 3’ ends of the two genes. The capsid and ORF3 genes of the two variants were almost identical (about 99% protein sequence identity), while their helicase genes were considerably divergent (53.4% protein sequence identity), explaining why there was twice the coverage of the initial mapping against this sequence for the Cap region as compared to the Rep region. Phylogenetic relationship to other known CRESS-DNA viruses, including the closely related human respiratory circovirus-like viruses (Genbank accession numbers KJ433989, KY214434, KY052047, KY349925, and KY244146) and members of the genus *Redondoviridae* [12], were studied using the whole-genome nucleotide sequences, and the Cap and Rep protein sequences. Maximum-likelihood phylogenetic analysis with the Rep protein sequences (since the Cap protein sequences had about 99% protein sequence identity) showed that the two HRCLVs were located within the vientoviruses branch (Figure 1). As shown in this figure, the variant that had a 99% identity with the sequence with an accession number of KY244146 was HRCLV-HULP2, while the HRCLV-HULP1 was farther away along the tree.

To explore the occurrence of HRCLV in patients with respiratory disease, we randomly selected one hundred anonymized DNA eluates extracted from clinical specimens from both upper and lower respiratory tracts obtained from patients at the HULP. In 54 of the samples, at least one infectious agent had been identified, while the remaining 46 were negative for all the pathogens tested. Respiratory syncytial virus, parainfluenza, and influenza A viruses were the most frequent in that set. Primers targeting the common and variant regions of HRCLV-HULP1 and HRCLV-HULP2 were designed (Table S1). qPCR was performed for these clinical samples, respiratory and serum samples obtained from the case patient, and a serum sample from the second patient (it was not possible to have other samples from the second patient since he was discharged at the time of this analysis and the serum sample was the only one stored). The qPCR reactions included the extraction negative controls and PCR negative controls that were, in all cases, negative. Two out of one hundred samples were found to be positive. One was positive for the two HRCLVs variants, for adenovirus and EBV, and the other was positive for HRCLV-HULP1 and for influenza A virus (H1N1). This shows that the two viruses detected in our patient sample are circulating among the local population and might not be related at all to travel. The serum samples from the two patients were negative. 

Although the link between these viruses and human disease is not established, recent work has shown that *Redondoviridae* may be associated with oropharyngeal and respiratory diseases [6], while other CRESS-DNA viruses are known animal pathogens, so their potential role as humans pathogens should be investigated in detail.

## Supplementary Materials

The following are available online at https://www.mdpi.com/1999-4915/12/3/327/s1, Table S1: Primer pairs used in this work.
