# Supplementary material for: Metagenomic Detection of Two Vientoviruses in a Human Sputum Sample
Source: Viruses. 2020 Mar 18;12(3):327. doi: 10.3390/v12030327 (PMC7150755; doi:10.3390/v12030327)
Supplement: Supplementary file 1 [file viruses-12-00327-s001.pdf]

Supplementary Materials

Table S1. Primer pairs used in this work.

Name	Forward Primer	Reverse Primer	Product Length (bp)
Cap 1	CTCTTACAACAGACCCAACTTC	CAAAGGCCTCTCTCCCTTCC	870
Cap 2	GGCTAAGCGATATGCATCAAG	ATAATTTCCTGATACATTAGC	777
Cap-Rep 1	CCTTCTGGGTCGTTATACG	CTCTCTTTCTTGATGCATATCG	290
Rep 1	CTGTTCTTCATCTTCGTGTG	GAGAAAGCACCCACCACG	758
Var 1-1	TCTACCGGTCTTGCTAATTG	GTACACCATCTGAAGTTGGG	1,417
Var 1-2	TGTAACATTCTATACCAAATGG	GCTTGCTAATAGTAGAAACAATAAG	1,196
Var 2-1	CAGGTTTGCTGTTTTTAGC	GTACACCATCTGAAGTTGGG	1,417
Var 2-2	TGTAACATTCTATACCAAATGG	AAGTAGAAGGAATTCTCCCTC	1,196
V1G1	ACACAGTCTAAACGTAGTGAC	TCTGATATTGTGAGCTTAGC	409
V1G2	GTGAAGGAGGCTAATGAGG	CAATTCTTTTACCGTTGTTAC	685
V2G1	GAATCCTTCTGGTTCAATG	ATCAAACGACTCCTTACG	431
V2G2	TATATTTGCTATTTGCTCTGC	CTTCTAATCTTCTTGACTTTCG	263
V2G3	ACAACATCAGATACTCCACC	GTTGATAAACCAGTAACACC	242
qPCR-Common	CTCTTACAACAGACCCAACTTC	TTCCTGATACATTAGCTTCTG	133
qPCR-Variant 1	TCTACCGGTCTTGCTAATTG	GCTTGCTAATAGTAGAAACAATAAG	273
qPCR-Variant 2	CAGGTTTGCTGTTTTTAGC	AAGTAGAAGGAATTCTCCCTC	254
